# Self-Supporting Three-Dimensional Electrospun Nanofibrous Membrane for Highly Efficient Air Filtration

**DOI:** 10.3390/nano11102567

**Published:** 2021-09-29

**Authors:** Gaofeng Zheng, Zungui Shao, Junyu Chen, Jiaxin Jiang, Ping Zhu, Xiang Wang, Wenwang Li, Yifang Liu

**Affiliations:** 1 Department of Instrumental and Electrical Engineering, Xiamen University, Xiamen 361102, China; 35120191151216@stu.xmu.edu.cn (Z.S.); 35120201151504@stu.xmu.edu.cn (J.C.); jiangjx@xmu.edu.cn (J.J.); 19920151153707@stu.xmu.edu.cn (P.Z.); 2 Shenzhen Research Institute of Xiamen University, Shenzhen 518000, China; 3 School of Mechanical and Automotive Engineering, Xiamen University of Technology, Xiamen 361024, China; wx@xmut.edu.cn (X.W.); xmlww@xmut.edu.cn (W.L.)

**Keywords:** air filtration, electrospun membrane, curled nanofibrous, self-supporting composite structure, three-dimensional pattern

## Abstract

High-performance air filtration was the key to health protection from biological and ultrafine dust pollution. A self-supporting, three-dimensional (3D) nanofibrous membrane with curled pattern was electrospun for the filtration, of which the micro-fluffy structure displayed high-filtration efficiency and low-pressure drop. The flow field in the 3D filtration membrane was simulated to optimize the process parameters to increase the filtration performance. The qualification factor increased from 0.0274 Pa^−1^ to 0.0309 Pa^−1^ by 12.77% after the optimization of the electrospinning parameters. The best filtration efficiency and pressure drop were 93.6% and 89.0 Pa, separately. This work provides a new strategy to fabricate 3D structures through the construction of fiber morphology and promotes further improvement of air filtration performance of fibrous filters.

## 1. Introduction

Public health and individual protection from environmental pollution has attracted more and more attention from all over the world [1,2,3]. At present, the biological and ultrafine dust pollution in the air has become a hot spot for economic development and human health [4,5,6]. Attributing to the properties of ultrafine feature size, high special surface area and porosity, nanofibrous membrane has good electrostatic absorption ability and displays great advantages for high-performance filtration [7,8,9]. However, fibers with a high packing density may cause high resistance that results in large energy loss, increasing the burden of the environment and hindering the realization of carbon neutrality. The fibrous membranes with high-filtration efficiency and low pressure are important factors for air purification, where energy consumption during the filtration process can be reduced at the same time [10,11,12].

Electrospinning is an effective micro-nanofiber fabrication technology, in which the viscoelastic solution is driven by an electric field force as a fine jet. The specific surface area and stereo structure of the micro-nanofiber membrane can be easily controlled by adjusting the electrospinning process parameters, which can be used to optimize the filtration performance and promote the application potential in the field of air purification [13,14,15]. Micro-nanofiber membranes with three-dimensional (3D) structures enable the optimization of the packing density and the porous structure inside the fibrous membrane, which provides an easy channel to reduce the friction resistance and pressure drop [16,17,18]. At present, the construction of 3D structures is mainly based on the composite structure with external support. Gao [9] et al. prepared a 3D composite membrane with polyacrylonitrile (PAN) microsphere structure by free surface electrospinning. The introduction of PAN microspheres enlarged the pores between the fibers and greatly reduced the pressure drop. Jiang [13] et al. constructed a 3D composite membrane with continuous electrospinning polyvinylidene fluoride (PVDF) nanofibers and PAN nanofibers. Thick PVDF nanofibers provided support to the 3D structure and optimized the packing density of the composite membrane, which reduced the pressure drop and improved the filtration performance. The overall structure of the fibrous membrane can be controlled by the processing parameters to achieve good filtration results. The effect of stereo fibrous morphology on the filtration performance should be further studied for industrial applications.

During the electrospinning process, the charged jet stepped into spiral instability motion due to the charge repulsion force. Besides the straight nanofiber, membranes with a curled nanofiber pattern can be gained by adjusting the processing parameters [19]. The curled nanofiber in the membrane will change the fluent field and rate distribution inside the membrane to increase the contact area between the air and nanofiber, enhance the electrostatic absorption—as the curled nanofibrous membrane has lower stacking density—and reduce the pressure drop. The spiral whipping motion of the electrospinning jet plays the key role in the formation of curled nanofibrous patterns [20,21,22], which can be produced easily by adjusting the process parameters. PVDF is an excellent candidate material for air filters for its properties of light weight, chemical stability and ease of processing [23,24,25]. Xiao [8] et al. produced a unique tree-like structure PVDF nanofiber by adding the silver nanoparticles with good filtration performance and high antibacterial activity. The filtration efficiency for 0.3 µm NaCl aerosol particles was 99.95–99.97% and the lowest pressure drop was 137.5 Pa. Liu [26] et al. fabricated self-powered electrostatic adsorption face masks by combining the PVDF nanofiber membrane and copper, resulting in long-term air filtration. The filtration efficiency for ultrafine particulates was 86.9 wt% and the average pressure drop was 170 Pa. Therefore, the optimization of fiber morphology is expected to further improve the performance of PVDF air filters.

In this paper, the curled PVDF electrospun microfiber was produced to establish a self-supporting 3D membrane, which can increase the porosity and reduce the pressure drop, owing to good air filtration performance. This new and easy fiber manufacturing strategy will further improve the air filtration performance of nanofibrous filters with high efficiency and low-pressure drop.

## 2. Experimental Details

### 2.1. Materials

Polyvinylidene fluoride (PVDF, *M*_w_ = 1,000,000 g/mol) was used to prepare the electrospun nanofibrous membrane for air filtration. PVDF powder was dissolved in a mixture solvent of dimethyl sulfoxide (DMSO) and acetone (*v*:*v* = 1:1) with a mass concentration of 10 wt% at 35 °C for 12 h. Polypropylene (PP) melt-blown nonwoven fabric was used as the substrate.

### 2.2. Preparation of Nanofibrous Membrane

**Figure 1:**
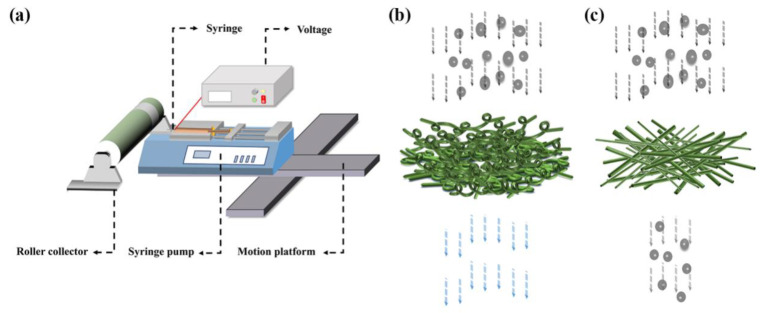
Schematic diagram of electrospinning setup and nanofibrous filtration membrane. (**a**) electrospinning setup; (**b**) air filtration process filtration membrane with curled PVDF nanofiber; (**c**) air filtration process filtration membrane with straight PVDF nanofibrous membrane.

The electrospinning process is shown in Figure 1a. The anode of the high-voltage source (DW-SA403-1ACE5, Dongwen High Voltage Power Supply Ltd. of Tianjin, Tianjin, China) was connected to the spinneret and the roller collector was grounded. The precision injection pump (Pump 11 Pico Plus Elite, Harvard Apparatus America, Holliston, MA, USA) was used to supply the polymer solution at a rate of 300 µL/h. The environmental temperature and relative humidity during the electrospinning process were controlled to be 24 °C and 35% RH, respectively. By adjusting the electrospinning parameters, two different morphologies of PVDF fiber were obtained, as shown in Figure 1b,c. In the experiments, The PVDF nanofibrous membrane was fabricated at distances between the spinneret (I. D. 200 µm, O. D. 410 µm) and the collector of 1.0 cm, 3.0 cm, 5.0 cm, 7.0 cm and 9.0 cm, separately. The applied voltage was 3 kV, 4 kV, 5 kV, 6 kV and 7 kV, separately. The rotation speed of the collector was 50 rpm, 200 rpm, 400 rpm, 600 rpm and 800 rpm, separately. The electrospinning time was set to 10 min, 30 min, 60 min, 90 min and 120 min, separately.

### 2.3. Characterization

#### 2.3.1. Morphology of PVDF Nanofibrous Membrane

The PVDF nanofibrous membrane was analyzed by the scanning electron microscopy (SEM, SU-70, Hitachi High-Technologies Corporation, Tokyo, Japan) under the voltage at 5 kV. ImageJ, an image processing software (1.52v, National Institutes of Health, Bethesda, Maryland, USA) was used to measure the microfiber diameter.

#### 2.3.2. Porosity Testing

The porosity (*PR*) was measured by the n-butanol absorption method, which can be measured as:

(1) $$PR=\frac{M_{1}-M_{2}}{\rho AH}$$

where $M_{1}$ and $M_{2}$ were the mass of PVDF nanofibrous membrane before and after soaking in an n-butanol solution, respectively. ρ was the density of the n-butanol solution. A and H were the area and thickness of PVDF nanofibrous membrane, respectively.

#### 2.3.3. Air Filtration Performance Testing

During the filtration testing, the sodium chloride (NaCl) aerosol particles with diameters of 0.3 µm passed through the PVDF nanofibrous membrane at an airflow of 80 L/min and the environmental temperature and humidity of 25 °C and 30 RH%. The filtration efficiency (FE) was calculated by the following equation:

(2) $$FE=\frac{N_{1}-N_{2}}{N_{1}}$$

where $N_{1}$ was the number of particles at the inlet and $N_{2}$ was the number of particles at the outlet of the testing channel to define the filtration efficiency, which was counted by two laser photometers. The filtration membrane with high-filtration efficiency and low-pressure drop was the goal for this work.

The quality factor (QF) was calculated to characterize the filtration performance of the PVDF nanofibrous membrane, which was obtained by the following equation:

(3) $$QF=-\frac{\ln\left(1-FE\right)}{\Delta P}$$

where ΔP was the pressure drop of the PVDF nanofibrous membrane, which was measured by a pressure transmitter.

#### 2.3.4. Air Filtration Behaviors Simulation

**Figure 2:**
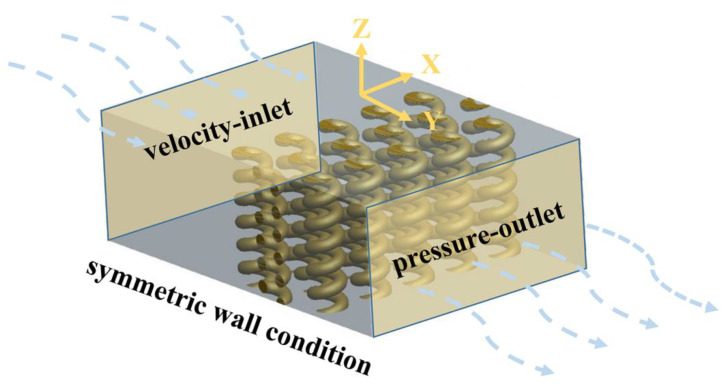
Simulation model of three-layer fibers.

The dynamic simulation analysis of the nanofiber model was operated in the software FLUENT (v11.0, ANSYS, Inc., Canonsburg, Pennsylvania, USA), as shown in Figure 2. The diameter of the fiber, the thickness of the fibrous membrane and the 3D structure of the fiber were determined according to different simulation aims. The cylindrical nanofibers with a diameter of 2.0 µm were arranged in three layers. As a special case of curled fiber, a spiral fiber model was structured to simulate the effects of curled nanofiber on air flow and particles. The helix radius of the three layers of nanofibers was 2.0 µm and the pitch was 2.1 µm, 2.5 µm, 3.0 µm, 3.5 µm and 4.0 µm, respectively. The spiral fiber coil diameter was 2 µm, the spacing between adjacent fibers in the same layer was 2.0 µm, the spacing between fibers in different layers was 2.0 µm and the multi-layer fibers were staggered. The pitch was defined as the distance between two adjacent threads measured along the helix direction. The spiral fiber coil diameter was defined as the diameter of the cylinder formed by the spiral fiber.

The air entered the calculation area from the velocity-inlet and flowed out of the calculation area from the pressure-outlet after passing through the fibrous filter. The empty areas 10/5 times larger than the fiber diameter were reserved between the inlet/outlet boundaries and the fiber surface, respectively, and the non-slip wall condition was used on the fiber surface. The airflow moved along the direction perpendicular to the thickness of the fibrous filter and the side of the calculation area was set as a symmetric wall condition. The velocity-inlet was set to be 0.5 m/s, the fiber diameter was selected as the characteristic length and the Reynolds number (Re) was defined as:

(4) $$Re=\frac{\rho vd}{\mu }$$

where ρ was the air density, μ was the dynamic viscosity, v was the air velocity and d was the characteristic length.

The model with steady state, laminar and incompressible flow was used in the simulation. Inertial particles were used in the simulation and the particles entered the calculation area as a surface jet source from the inlet. In addition, in the electrospinning process, once the particles collided with the fibrous filter, they were considered to be trapped and would not rebound again. The particles were considered to escape when they left the filter. The calculation area was divided by a tetrahedral grid, the density of particles was 1800 kg/m^3^, the diameter of particles was 0.5 µm and the inlet mass flow was 5 × 10^−8^ kg/s. Additionally, the DPM model was used for simulation analysis. When the particles passed through the fibrous filter, the effects of inertia and diffusion caused by Brownian motion on the filtration efficiency were mainly considered, while the interaction between the particles and the influence of the electric field force and gravity on the particles were ignored. The diffusion efficiency ($\eta_{D}$) was obtained by the following equations:

(5) $$\eta_{D}=2.6{\left(\frac{1-\propto }{Ku}\right)}^{\frac{1}{3}}Pe^{-\frac{2}{3}}$$

(6) $$Pe=\frac{v_{0}d_{f}}{D}$$

(7) $$D=\frac{KTC_{u}}{3\pi \mu d_{p}}$$

where ∝ was the filling rate, *D* was the diffusion coefficient of the particles, $v_{0}$ was the airflow velocity, K was Boltzmann constant, u was fluid velocity, T was absolute temperature, $C_{u}$ was sliding correction coefficient, μ was gas viscosity, $d_{p}$ was particle diameter and $d_{f}$ was fiber diameter.

## 3. Results and Discussion

### 3.1. Dynamic Simulation of Air Flow Process

**Figure 3:**
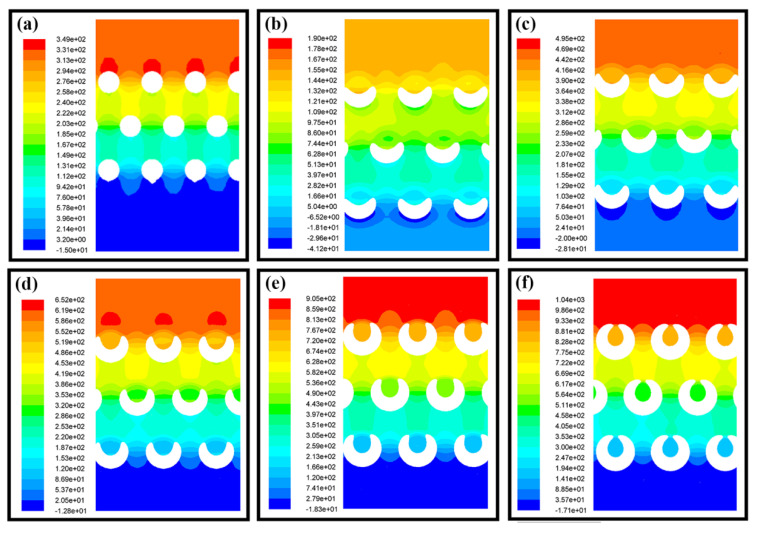
The static pressure distribution of the airflow inside different nanofibrous membrane layers. (**a**) cylindrical nanofiber with a diameter of 2.0 µm; (**b**–**f**) spiral nanofibers with a diameter of 2.0 µm. The pitch was 4.0 µm, 3.5 µm, 3.0 µm, 2.5 µm and 2.1 µm in (**b**–**f**), respectively.

The static pressure distributions of the airflow inside different nanofiber membranes at the same height are shown in Figure 3. As shown in Figure 3a, for the cylindrical nanofibers, the airflow pressure decreased layer by layer and the pressure on the windward side was much higher than that on the leeward side. Additionally, the air pressure distribution between the nanofiber layers was not uniform, showing a well-defined vertical distribution. Similar to cylindrical nanofibers, when the pitches of spiral nanofibers were large, the pressure on the windward side was also higher than that on the leeward side, as shown in Figure 3b–d. However, the air pressure distribution between the layers of spiral nanofibers was more uniform than that of cylindrical fibers, presenting a uniformly distributed low pressure, which was a good way to improve the interception effect. Nevertheless, with the decrease of the spiral fiber pitch, the fibers tend to be cylindrical, which made the pressure distribution inside the nanofibers more similar to that of cylindrical fibers, as shown in Figure 3e,f. Therefore, the nanofiber shape should be controlled and optimized to ensure a proper pitch.

**Figure 4:**
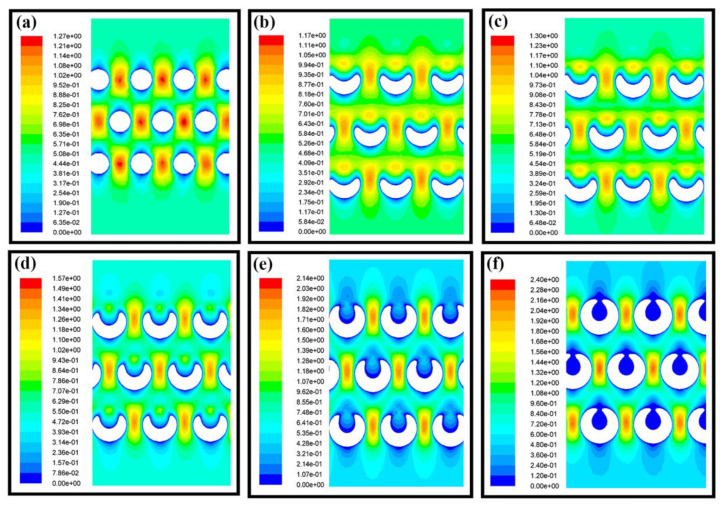
The velocity distribution of airflow in different fiber membranes. (**a**) cylindrical fiber with a diameter of 2.0 µm; (**b**–**f**) spiral fibers with a diameter of 2.0 µm and the pitch was 4.0 µm, 3.5 µm, 3.0 µm, 2.5 µm and 2.1 µm, respectively.

The airflow field in the filtration membrane was the main role to define the motion trajectory and the velocity, which have a great influence on the air filtration process. For the cylindrical nanofibers, the velocity of the airflow increased greatly due to the turbulence and extrusion between the fibers in the same layer, as shown in Figure 4a. However, due to the existence of hollow gaps in the spiral fibers, the path of airflow was changed and the squeezing effect of the airflow between the fibers was not obvious so that the airflow tended to be evenly distributed and the velocity was reduced, as shown in Figure 4b–d. Therefore, the diffusion effect of the spiral fiber was improved compared with the cylindrical fibers, which was also the main air filtration mechanism of the fiber filters. With the decrease of the spiral fiber pitch, the fibers tend to be cylindrical, which also made the velocity distribution inside the fibers more similar to that of cylindrical fibers, as shown in Figure 4e,f. Thus, spiral fibers could produce a special flow field compared with the cylindrical fiber, which was helpful to improve the air filtration performance. However, it was still necessary to further regulate the morphology of spiral fibers in order to achieve better air filtration performance.

### 3.2. Air Filtration of PVDF Nanofibrous Membrane

Processing parameters of electrospinning had a key influence on the morphology of electrospun fibers. Optimizing electrospinning parameters to gain the 3D structure of the fibers was very helpful to the improvement of air filtration performance.

**Figure 5:**
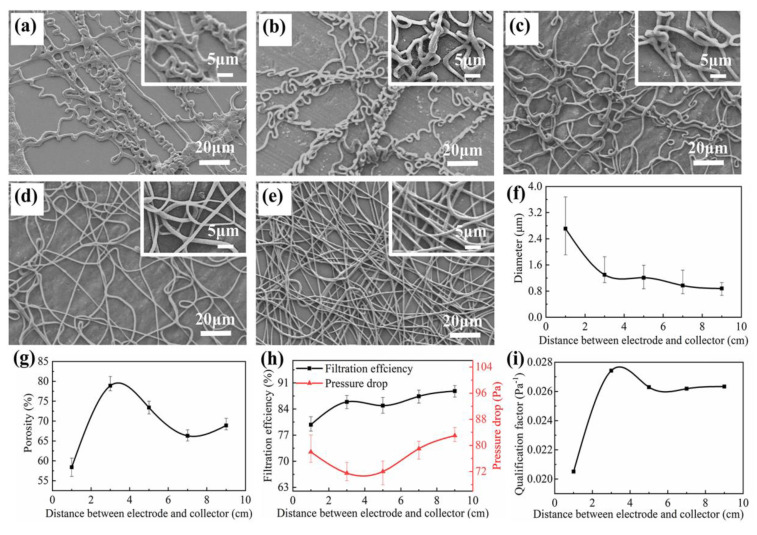
The SEM image of nanofibrous membrane and filtration performance with different distance between electrode and collector. (**a**–**e**) SEM image of PVDF nanofibrous membrane at the distance between electrode and collector of 1.0 cm, 3.0 cm, 5.0 cm, 7.0 cm and 9.0 cm, respectively; (**f**–**i**) diameter, porosity, filtration efficiency and pressure drop, *QF* of PVDF nanofibrous membrane at different distance between electrode and collector, respectively. The voltage, the roller speed and the electrospinning time was 5 kV, 50 rpm and 60 min, respectively.

The stretching degree of the PVDF microfibers increased with the distance between electrode and collector, as shown in Figure 5a–e. When the distance was 1.0 cm, the solvent did not volatilize completely and the PVDF microfibers were not fully stretched, resulting in a small amount of liquid film. With the gradual increase of the distance, curled PVDF microfibers appeared due to the second-level bending instability of the curved fiber regions. The microfibers tended to be straight if the distance kept rising. Finally, the fiber was fully stretched when the distance was raised to 9.0 cm and all PVDF microfibers became straight. With the increase of distance, the average diameter of PVDF microfibers decreased and the uniformity increased, as shown in Figure 5f. The change of distance affected the morphology of the PVDF microfibers which affected the porosity of the PVDF nanofibrous membrane. When the distance was 3.0 cm, there were more curled fibers and the porosity reached the maximum value of 78.9%. The curled fibers disappeared and the porosity decreased for the distance of 7.0 cm. As the distance was further increased, the decrease of fiber diameter made the porosity rebound, as shown in Figure 5g. The results of filtration efficiency and pressure drop are shown in Figure 5h. As the distance increased from 1.0 cm to 3.0 cm, the diameter of the fiber decreased and the specific surface area increased, which increased the filtration efficiency of the fibrous filter. At the same time, the appearance of curled fiber increased the porosity and that decreased the pressure drop. With the further increase of distance, the curled fibers disappeared, the PVDF microfibers stacked tightly and the filtration efficiency and pressure drop of the fibrous membrane increased. Therefore, the *QF* of the PVDF nanofibrous membrane increased at a distance between electrode and collector from 1.0 cm to 3.0 cm, decreased from 3.0 cm to 9.0 cm and had the best *QF* of 0.0274 Pa^−1^ at 3.0 cm, as shown in Figure 5i, which had more curled PVDF nanofibers in the filtration membrane.

**Figure 6:**
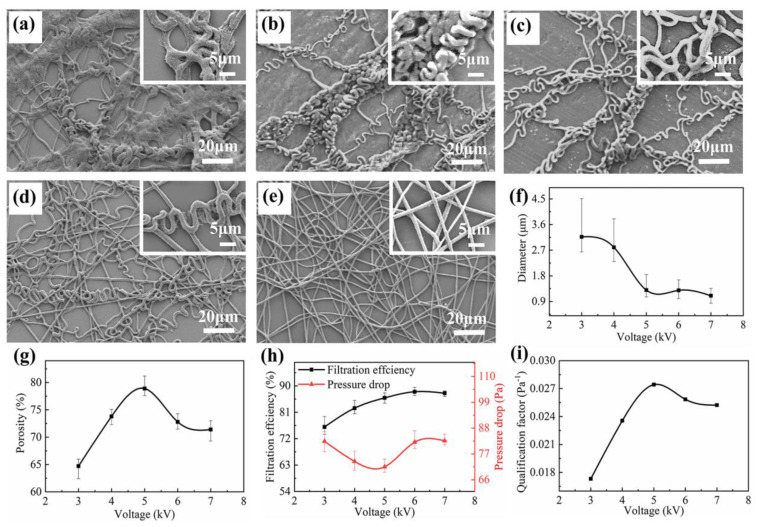
The SEM image, nanofiber diameter and filtration performance of nanofibrous membrane with different applied voltage. (**a**–**e**) SEM image of PVDF nanofibrous membrane at the voltage of 3.0 kV, 4.0 kV, 5.0 kV, 6.0 kV and 7.0 kV, respectively; (**f**–**i**) diameter, porosity, filtration efficiency and pressure drop, *QF* of PVDF nanofibrous membrane at different voltage, respectively. The distance between electrode and collector, the roller speed and the electrospinning time was 3.0 cm, 50 rpm and 60 min, respectively.

The applied voltage was the energy sources for the injection of the electrospinning jet, which was the key role in the whipping state of the electrospinning jet and nanofiber morphology. Thus, the morphology and deposition pattern of the nanofibrous membrane can be adjusted with varying voltages. As shown in Figure 6a–e, with the increase of the voltage, the degree of the screw whipping of the fibers becomes larger leading to straight nanofiber structures. The average diameter of the PVDF microfibers decreased with the increase of voltage, which reflected the tensile effect of the voltage on the fibers, as shown in Figure 6f. In addition, with the increase of voltage, the fluffy degree of the PVDF nanofibrous membrane changed. As shown in Figure 6g, when the voltage increased from 3 kV to 5 kV, the winding tightness of the curled fibers decreased, resulting in the increase of the porosity. With the further increase of the voltage, the spiral fiber disappeared and the PVDF nanofibrous membrane stacked tightly, resulting in the decrease of the porosity. The filtration efficiency and pressure drop of the PVDF nanofibrous membrane with different voltage were shown in Figure 6h. With the increase of the voltage, the diameter of the PVDF microfibers decreased, the specific surface area of the PVDF microfibers increased, thus the filtration efficiency increased. When the voltage was greater than 5 kV, the curled PVDF microfibers gradually disappeared and the fibers tightly stacked and the resistance to the airflow increased, resulting in the rising of the pressure drop. Therefore, when the voltage was 5 kV, *QF* reached the highest value of 0.0274 Pa^−1^, as shown in Figure 6i.

**Figure 7:**
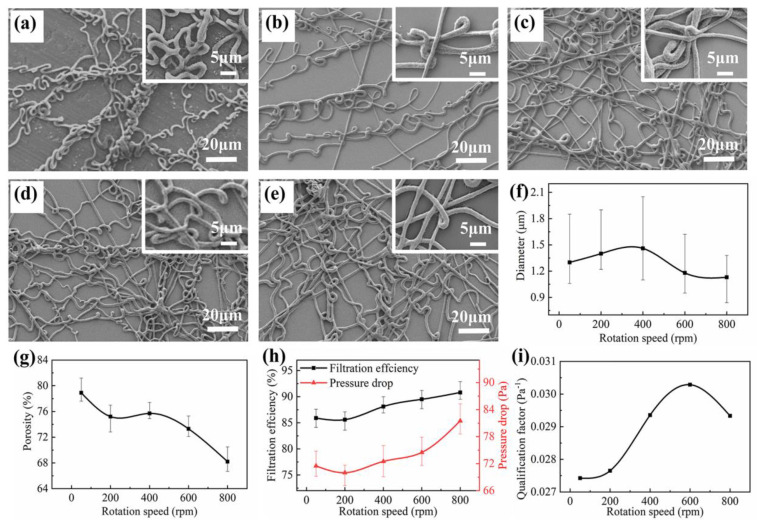
The SEM image, nanofiber diameter and filtration performance of nanofibrous membrane with roller speed. (**a**–**e**) SEM image of PVDF nanofibrous membrane at the roller speed of 50 rpm, 200 rpm, 400 rpm, 600 rpm and 800 rpm, respectively; (**f**–**i**) diameter, porosity, filtration efficiency and pressure drop, *QF* of PVDF nanofibrous membrane at different roller speeds, respectively. The distance between electrode and collector, the voltage and the electrospinning time was 3.0 cm, 5 kV and 60 min, respectively.

Since the roller collector was dragging the nanofiber during the decomposition process, the rotation speed of the roller collector was also an important factor defining the deposition morphology of the electrospun nanofibers. As the rotation speed of the roller increased, the PVDF microfibers tended to be straight while the average diameter remained almost unchanged, as depicted in Figure 7a–f. The porosity of the PVDF nanofibrous membrane increased with the rotation speed because of the tight stacking of straight PVDF microfibers, as shown in Figure 7g. The filtration efficiency and pressure drop of the PVDF nanofibrous membrane were shown in Figure 7h. When the rotation speed increased from 50 rpm to 200 rpm, the PVDF nanofibrous membrane had a higher porosity that resulted in lower filtration efficiency and pressure drop. With further increase of the rotation speed, the PVDF microfibers stacked tightly, the porosity was reduced, thereby improving the filtration efficiency and increasing the pressure drop. In particular, when the rotational speed was further increased, the PVDF microfibers stacked more closely and the porosity decreased significantly, resulting in a sharp increase in pressure drop. A maximum *QF* of 0.03029 Pa^−1^ was found at the roller speed of 600 rpm, as shown in Figure 7i.

**Figure 8:**
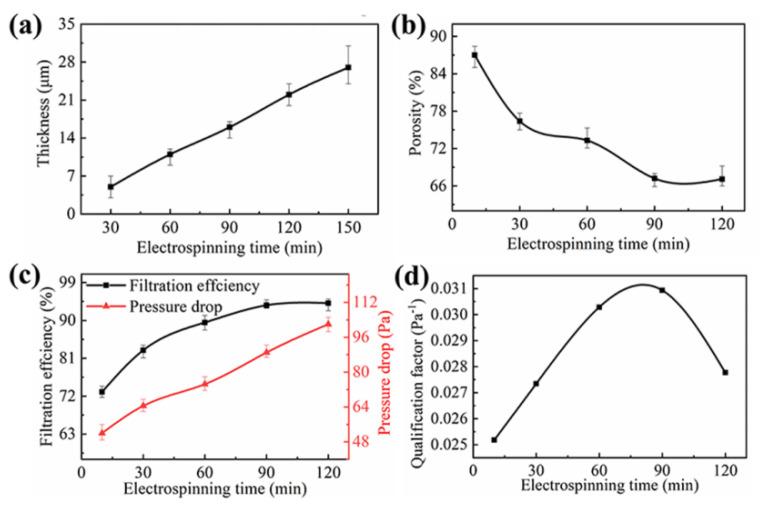
(**a**) The thickness of PVDF nanofiber membrane with electrospinning time. (**b**–**d**) The porosity, filtration efficiency and pressure drop, *QF* of PVDF nanofibrous membrane at different electrospinning time, respectively. The distances between electrode and collector, the voltage and the roller speed were 3.0 cm, 5 kV and 600 rpm, respectively.

The processing parameter was optimized. The distance between the electrode and the collector, the applied voltage and the roller speed of 3.0 cm, 5 kV and 600 rpm, respectively, were determined as the best electrospinning parameters to explore the thickness of the PVDF nanofibrous membrane with the best filtration performance. The variation of the electrospinning time resulted in the variation of the thickness of membrane. The thickness of membrane increased linearly with the increase of electrospinning time from 5 µm to 27 µm, as shown in Figure 8a. As shown in Figure 8b,c, with the electrospinning time increased to 60 min, the PVDF microfibers continued to deposit and, thus, the porosity, the filtration efficiency and the pressure drop increased concurrently. When the electrospinning time was further increased, the filtration efficiency and pressure drop continued to rise, while the PVDF microfibers stacked more tightly and the porosity decreased sharply. The *QF* reached the highest value of 0.0309 Pa^−1^ at the electrospinning time of 90 min with the best filtration efficiency and pressure drop of 93.6% and 89.0 Pa, respectively, as shown in Figure 8d.

## 4. Conclusions

The nanofibrous membrane with curled PVDF nanofibrous pattern was fabricated through the electrospinning process. The self-supporting 3D curled structure was a good way to improve the filtration performance. The airflow behaviors in the air filtration with curled PVDF nanofiber were simulated to study the flow-field distribution. Furthermore, the electrospinning parameters were optimized to further improve the filtration performance of the PVDF nanofibrous membrane. The results show that the curled PVDF microfibers had the self-supporting effect to construct 3D composite structure. The optimized curled PVDF nanofibrous membrane had better filtration performance than the straight fiber. In the curled nanofibrous membrane, the *QF* increased by 12.77% through optimization of electrospinning parameters. This work provides a new way to produce 3D nanofibrous membrane filtration structures, which show better air filtration performance with high efficiency and low-pressure drop.

## Data Availability

The data presented in this study are available on request from the corresponding author.
